# Low whole blood selenium level is associated with higher mortality and longer ICU and hospital stay in patients undergoing elective cardiac surgery

**DOI:** 10.1186/cc9793

**Published:** 2011-03-11

**Authors:** G Koszta, Z Kacska, K Szatmári, T Szerafin, C Mitre, B Fülesdi

**Affiliations:** 1 University of Debrecen, Hungary; 2 University of Cluj, Romania

## Introduction

It has been shown that low selenium intake is a risk factor for mortality in several diseases and conditions. In the present study we assessed the association between preoperative selenium levels and outcome parameters in patients undergoing elective cardiac surgical procedures.

## Methods

Whole blood selenium levels were assessed in preoperatively sampled blood in 197 patients. Selenium levels were dichotomized according to the national reference values into low (<100 μg/l = LS group) and normal (>100 μg/l = NS group). Preoperative risk factors and postoperative outcome parameters (such as mortality, ICU and hospital length of stay, postoperative complications) were compared among the two selenium groups.

## Results

The mean age of the patients in the LS group was 67.9 ± 8.9 years, significantly higher than the NS group's mean age of 62.05 ± 9.4 years (*P *< 0.01). The mean EuroSCORE was 0.0560 ± 0.069 in the LS group, while it was 0.1071 ± 0.1192 in the NS group (*P *< 0.01). The relative risk of mortality in the LS group was 5.01. The ICU length of stay was longer in the LS group (4.55 ± 7.1 days) compared with the NS group (2.54 ± 4.5 days, *P *< 0.01). Similar to this, the hospital length of stay was also longer in the LS group (12.46 ± 10.4 days) than in the NS group (8.44 ± 4.81 days, *P *< 0.01). LS patients were more frequently presented in the postoperative phase with low cardiac output syndrome, atrial fibrillation, postoperative renal failure and postoperative confusion.

## Conclusions

We conclude that low selenium levels are associated with higher mortality and longer hospital stay in our Central-European cohort of cardiac surgical patients. Prospective randomized studies performed on homogeneous patient groups are encouraged to prove whether the postoperative outcome of the patients may be improved by preoperative normalisation of selenium levels.

